# Draft genome of the switchgrass head smut pathogen *Tilletia maclaganii*

**DOI:** 10.1128/mra.00991-25

**Published:** 2025-11-05

**Authors:** Gian Maria Niccolò Benucci, Kevin L. Myers, Chathu Wijewardana, Gary C. Bergstrom, Stephen Mondo, Robert Riley, Anna Lipzen, Jonathan Del Rosario, Mi Yan, Vivian Ng, Igor V. Grigoriev, Acer VanWallendael, David B. Lowry

**Affiliations:** 1 Department of Plant, Soil and Microbial Sciences, Michigan State University 242456, East Lansing, Michigan, USA; 2 Great Lakes Bioenergy Research Center (GLBRC) 282101, East Lansing, Michigan, USA; 3 School of Integrative Plant Science, Plant Pathology and Plant-Microbe Biology Section, Cornell University 517685, Ithaca, New York, USA; 4 U.S. Department of Energy Joint Genome Institute (JGI), Lawrence Berkeley National Laboratory 1666, Berkeley, California, USA; 5 Department of Plant and Microbial Biology, University of California Berkeley 118549, Berkeley, California, USA; 6 Department of Plant Biology, Michigan State University 124535, East Lansing, Michigan, USA; 7 Plant Resilience Institute, Michigan State University 3078, East Lansing, Michigan, USA; 8 Department of Horticultural Science, North Carolina State University 228326, Raleigh, North Carolina, USA; 9 Department of Crop and Soil Science, North Carolina State University 242502, Raleigh, North Carolina, USA; Rochester Institute of Technology, Rochester, New York, USA

**Keywords:** basidiomycota, Ustilaginales, *Ustilago*, switchgrass head smut, *Panicum virgatum*, PacBio

## Abstract

*Tilletia maclaganii* is a smut fungal pathogen that causes significant biomass reduction of switchgrass (*Panicum virgatum*) used for animal forage and biofuel production. Here we present the annotated genome of *T. maclaganii*, strain Tm001-NY21, estimated at 42.79 Mb in size, in 53 assembled contigs and encoding 10,235 predicted genes. This genome will be important for future comparative studies of Ustilaginales across its geographic and host range.

## ANNOUNCEMENT

*Tilletia* is a genus of biotrophic smut fungi (Tilletiaceae, Ustilaginales) that parasitize grasses by forming teliospores in host reproductive tissues (1). Some species, such as *Tilletia caries* and *Tilletia indica*, are major wheat pathogens of economic and quarantine concern (2, 3). *Tilletia maclaganii* (Berk.) G.P. Clinton (4), originally described from South American grasses, is an emerging pathogen of switchgrass (*Panicum virgatum*) that reduces biomass and affects its use as forage, biofuel feedstock, and ornamental plants (5, 6). Genomic analysis of *T. maclaganii* informs host specialization and smut fungal diversity and serves as a reference for population genomics.

*T. maclaganii* Tm001-NY21 was isolated from a single basidiospore on switchgrass cv. Shawnee in Ithaca, NY, USA (42.4654130 N, −76.4590049 W) in 2021. Mycelium was cultured in Malt Extract Peptone Broth for 14 days at 24°C. DNA was extracted from 1.5 g ground fungal tissue in liquid nitrogen with the Macherey-Nagel Nucleobond HMW kit. For RNA, the fungus was treated with 1 ppm pentachloronitrobenzene prior to extraction using the Monarch Total RNA Miniprep Kit (NEB, MA).

DNA was sequenced on a PacBio Sequel IIe using ~10 kb sheared DNA with SMRTbell adapters, size-selected via BluePippin (Sage Science, MA), and processed using PacBio protocols. RNA was sequenced on an Illumina NovaSeq 6000 with TruSeq Stranded mRNA prep (poly-A selection) from 1 µg RNA/sample, using eight PCR cycles and 2 × 150 bp indexed runs. A total of 548,060 DNA and 181.18 M RNA-Seq total reads were filtered with *bbduk.sh* (BBTools v38.79) to remove low-quality reads. Filtered DNA reads were assembled with Flye v2.9-b1768 (7) and polished with RACON v1.4.13 (8) twice. Filtered RNA-Seq reads were assembled using Trinity v2.11.0 (9). Genome annotation was performed with the JGI Annotation Pipeline using Illumina transcriptome data (10, 11). Default parameters were used unless stated otherwise.

*T. maclaganii* Tm001-NY21 genome is estimated at 42.79 Mbp with 143.3× sequence coverage, 53 contigs, N50 = 1.62 Mbp (length where contigs ≥ this size sum to half the genome); L50 = 11 (number of contigs needed to reach half the genome), and GC content = 58.2%. The assembly had 97% completeness by BUSCO (basidiomycota_odb10) and captured 94.6% of RNA-seq (12). The mitochondrial genome spans 265,525 bp across five contigs (N50 = 78,073 kb; L50 = 2). The genome of *T. maclaganii* is larger than those of *T. indica* (30.32 Mbp), *T. caries* (29.54 Mbp), and *T. controversa* (28.84 Mbp). A total of 10,235 protein-coding genes were predicted, averaging 2,103 bp (521 bp for proteins), similar to *T. caries* (10,204), *T. controversa* (9,860), and *T. indica* (9,548).

## Data Availability

Whole-genome and transcriptome raw reads are available in the SRA under accession SRP602000 (NCBI: PRJNA1080998), and SRP602007, SRP602006 (NCBI: PRJNA1287810 and PRJNA1287828), respectively. The genome assembly and annotations are in GenBank under accession JBQVXD000000000 and at the JGI MycoCosm portal (10) https://mycocosm.jgi.doe.gov/Tilmac1. Tilletia maclaganii Tm001-NY21 live culture is available at NRRL 64469 (USDA-ARS-NCAUR, Peoria, IL), and a symptomatic switchgrass specimen is archived as CUP-073118 in the Cornell University Plant Pathology Herbarium. Detailed DNA/RNA and bioinformatics methods are available at protocols.io: https://dx.doi.org/10.17504/protocols.io.dm6gpmr55gzp/v2.
